# Long-Term Effects of Postoperative Atrial Fibrillation following Mitral Valve Surgery

**DOI:** 10.3390/jcdd10070302

**Published:** 2023-07-16

**Authors:** Haneen Alghosoon, Amr A. Arafat, Monirah A. Albabtain, Faisal F. Alsubaie, Abdulaziz S. Alangari

**Affiliations:** 1 Research Department, Prince Sultan Cardiac Center, Riyadh 12233, Saudi Arabia; halghasoon@pscc.med.sa (H.A.); muneera_2004@yahoo.com (M.A.A.); 2 Department of Epidemiology and Biostatistics, College of Public Health and Health Informatics, King Saud bin Abdulaziz University for Health Sciences, Riyadh 11481, Saudi Arabia; angaria@ksau-hs.edu.sa; 3 Adult Cardiac Surgery Department, Prince Sultan Cardiac Center, Riyadh 12233, Saudi Arabia; 4 Cardiothoracic Surgery Department, Tanta University, Tanta 31111, Egypt; 5 Respiratory Therapy Dept., Prince Sultan Cardiac Center, Riyadh 12233, Saudi Arabia; faisal-rt@hotmail.com; 6 King Abdullah International Medical Research Center, Riyadh 11481, Saudi Arabia

**Keywords:** postoperative atrial fibrillation, mitral valve replacement, mitral valve repair, short- and long-term outcomes

## Abstract

**Simple Summary:**

The incidence of postoperative atrial fibrillation (PoAF) after mitral valve surgery has not been thoroughly evaluated. This study evaluated postoperative atrial fibrillation incidence and its short- and long-term effects in patients who underwent mitral valve surgery. The overall incidence of PoAF was 16.8%. PoAF was associated with higher operative mortality, stroke, and dialysis. ICU and hospital stays were significantly longer in patients with PoAF. PoAF was significantly associated with increased mortality risk, heart failure rehospitalization, and stroke. The study revealed that atrial fibrillation after mitral valve surgery is common. PoAF was associated with an increased risk of short- and long-term adverse events. Future studies are needed to evaluate strategies that can be implemented to improve the outcomes of these patients. These strategies include the use of preventive antiarrhythmic therapy and close follow-up of those patients.

**Abstract:**

Background: New-onset postoperative atrial fibrillation (PoAF) is one of the most frequent yet serious complications following cardiac surgery. Long-term consequences have not been thoroughly investigated, and studies have included different cardiac operations. The objectives were to report the incidence and short- and long-term outcomes in patients with PoAF after mitral valve surgery. Methods: This is a retrospective cohort study of 1401 patients who underwent mitral valve surgery from 2009 to 2020. Patients were grouped according to the occurrence of PoAF (*n* = 236) and the nonoccurrence of PoAF (*n* = 1165). Long-term outcomes included mortality, heart failure rehospitalization, stroke, and mitral valve reinterventions. Results: The overall incidence of PoAF was 16.8%. PoAF was associated with higher rates of operative mortality (8.9% vs. 3.3%, *p* < 0.001), stroke (6.9% vs. 1.5%, *p* < 0.001), and dialysis (13.6% vs. 3.5%, *p* < 0.001). ICU and hospital stays were significantly longer in patients with PoAF (*p* < 0.001 for both). PoAF was significantly associated with an increased risk of mortality [HR: 1.613 (95% CI: 1.048–2.483); *p* = 0.03], heart failure rehospitalization [HR: 2.156 (95% CI: 1.276–3.642); *p* = 0.004], and stroke [HR: 2.722 (95% CI: 1.321–5.607); *p* = 0.007]. However, PoAF was not associated with increased mitral valve reinterventions [HR: 0.938 (95% CI: 0.422–2.087); *p* = 0.875]. Conclusions: Atrial fibrillation after mitral valve surgery is a common complication, with an increased risk of operative mortality. PoAF was associated with lower long-term survival, increased heart failure rehospitalization, and stroke risk. Future studies are needed to evaluate strategies that can be implemented to improve the outcomes of these patients.

## 1. Introduction

New-onset postoperative atrial fibrillation (PoAF) is one of the most common and serious complications of cardiac surgeries [1,2]. PoAF increases short-term morbidity and mortality, which causes an economic burden because of the prolonged intensive care unit (ICU) and hospital stay [3]. PoAF is estimated to be linked to considerable financial burdens on the healthcare system, with estimated yearly expenses between $6 and $26 billion in the United States alone [4]. Several adverse events have been associated with PoAF, including an increased risk of stroke, hemodynamic instability [5], and poor long-term survival [6].

Most studies evaluating PoAF have focused on coronary artery bypass grafting (CABG); additionally, the long-term effects of PoAF have not been thoroughly evaluated [7]. Rheumatic fever is still prevalent in our region; as a result, mitral valve surgery remains the main cardiac surgery performed [8,9,10]. Valvular surgeries lead to more damage to the heart’s structural integrity than CABG, leading to a potentially higher risk of developing PoAF [11].

The incidence of PoAF varies widely in the literature and ranges from 20 to 40%; moreover, the exact mechanism of pathophysiology has yet to be fully understood [11,12]. Although PoAF has been extensively researched in the past twenty years, advances in surgical care have not reduced the incidence of the associated complications arising from PoAF [6]. Furthermore, the long-term sequences of PoAF following mitral valve surgery have not been thoroughly evaluated. The objectives were to report the incidence of PoAF following mitral valve surgery and to evaluate the long-term effects of PoAF on survival, heart failure rehospitalization, stroke, and mitral valve reinterventions.

## 2. Patients and Methods

### 2.1. Study Design and Sample

This research is a retrospective cohort study of 1401 patients who underwent mitral valve surgery (repair or replacement), including isolated, concomitant, primary, or reoperative procedures, in a single tertiary referral center from January 2009 to January 2020. The exclusion criteria were as follows: (1) patients with a history of preoperative atrial fibrillation, (2) patients with missing data on preoperative rhythm, and (3) patients with preoperative implantable defibrillators or permanent pacemakers. Patients were grouped according to the occurrence of PoAF (*n* = 236) and the nonoccurrence of PoAF (*n* = 1165). The study was approved by the Institutional Review Board (IRB) (Approval Number: 1650). Given the retrospective nature of the investigation, the IRB approved the data collection and waived the requirement for the patient’s consent.

### 2.2. Data Collection and Measures

Paper records and surgical reports were reviewed in addition to computerized patient charts to obtain the clinical data needed for this research. Preoperative and baseline data that were collected included age, gender, cardiovascular risk factors such as diabetes mellitus, extracardiac arteriopathy, poor mobility, chronic lung disease, history of endocarditis, history of arrhythmia, and previous myocardial infarction. A history of previous cardiac surgery was reported. Patient symptoms such as shortness of breath were collected according to the New York Heart Association (NYHA) functional classifications, and critical preoperative status defined by the presence of preoperative ventilation, inotropic support, and the presence of an intra-aortic balloon pump (IABP) was also obtained. CHA₂DS₂-VASc was calculated from the following variables: age, sex, congestive heart failure history, hypertension, stroke/transient ischemic attack, vascular disease, and diabetes mellitus [13]. Perioperative data were retrieved, including surgery date, type, urgency, cardiopulmonary bypass, and aortic cross-clamp times. Early outcomes included PoAF, stroke, need for IABP or extracorporeal membrane oxygenation (ECMO), and severe renal impairment requiring dialysis; additionally, ICU and postprocedure hospital stays were reported. PoAF was defined as any episode of atrial fibrillation that occurred postoperatively during hospitalization and required treatment. The diagnosis of PoAF was made through continuous cardiac monitoring and confirmed with a 12-lead ECG.

### 2.3. Follow-Up

All patients were clinically followed-up at the institution’s outpatient clinic at 6 and 12 months and at yearly intervals after surgery. The most recent information available in the electronic medical record was obtained and included in the follow-up analysis. The close follow-up date was December 2022.

### 2.4. Study Endpoints

Study endpoints were early and late postoperative outcomes. Early outcomes include in-hospital mortality, stroke, and acute renal failure requiring dialysis or continuous renal replacement therapy. Late outcomes included survival, stroke, heart failure rehospitalization, and mitral valve reinterventions.

### 2.5. Statistical Analysis

#### 2.5.1. Data Presentation

The quantitative descriptive analysis method was used to describe the study sample. The Shapiro-Wilk test was used to determine the normality of the distribution of continuous data, and normally distributed variables were reported as the means ± standard deviations and were compared using an independent samples t test. Nonnormally distributed data were reported as medians (25th and 75th percentiles) and were compared using the Mann-Whitney U test. Categorical data are presented as counts and percentages. Comparison of categorical data was performed using Pearson’s chi-squared test, but if the expected frequency was less than 5, Fisher’s exact test was used.

#### 2.5.2. Time-to-Event Analysis

Survival and freedom from late stroke, heart failure rehospitalization, and mitral valve reinterventions were graphically plotted using the Kaplan–Meier method. The log-rank test was used to compare time-to-event outcomes between patients with and without PoAF. The occurrence of the event (death, stroke, rehospitalization, or mitral valve reinterventions) was analyzed by censoring patients at their time of death or last follow-up, and the time analyzed was between the surgery and occurrence of the event or at the time of a patient’s last follow-up. Multivariable Cox regression analysis was used to determine factors associated with time-to-event outcomes. A stepwise forward selection was used, and variables with a *p* value of less than 0.05 were retained in the final model. PoAF was included in the final model, even if not significant.

Statistical significance was defined as a *p* value of less than 0.05. Statistical analysis was performed utilizing IBM Statistical Package for Social Sciences (SPSS) version 29.0 (IBM Corp, Chicago, IL, USA) and Stata 17 (Stata Corp, College Station, TX, USA).

## 3. Results

### 3.1. Baseline Characteristics and Operative Data

During the study period, a total of 2175 patients underwent isolated or concomitant mitral valve surgery. Following the exclusion criteria, 523 (24.04%) patients with preoperative atrial fibrillation (AF) and 102 (4.69%) and 149 (6.85%) patients with missing preoperative or postoperative AF status were all excluded.

The final analysis included 1401 patients. The patients’ demographics and baseline characteristics are described and compared in Table 1. Patients who developed new-onset PoAF were significantly older [62 (25th–75th (49.5–70) vs. 55 (43–63) years, *p* < 0.001). Preoperative creatinine in patients with PoAF was significantly higher [81 (67–112) vs. 77 (62–93) μmol/L, *p* < 0.001) and more likely to be on dialysis (6.4% vs. 2.8%, *p* = 0.005). Additionally, patients with PoAF were more likely to have a critical preoperative state requiring an intra-aortic balloon pump (IABP) (*p* < 0.001), inotropic support (*p* < 0.001), and mechanical ventilation (*p* = 0.002) prior to cardiac surgery. Patients with PoAF had significantly higher EuroSCORE [3.4 (1.4–7.5) vs. 2.4 (1.3–5.2); *p* < 0.001]. There was no significant difference between groups regarding smoking history, diabetes mellitus, hypertension, previous stroke, and chronic obstructive lung disease. The preoperative CHA₂DS₂-VASc score was significantly higher in patients with PoAF [2 (1–4) vs. 2 (1–3); *p* = 0.003].

“Patients who developed PoAF were more likely to be operated on in emergency status (5.5% vs. 2.3%, *p* = 0.26), had mitral valve replacement surgery (54% vs. 46.2%, *p* = 0.029), and had significantly longer aortic cross-clamp time [109 (78–133) vs. 100 (75–126) minutes, *p* = 0.025). There was no difference in PoAF between patients who had mechanical vs. bioprosthetic valves [37 (15.7%) vs. 90 (21.1%); *p* = 0.091].” (Table 2).

### 3.2. Incidence of New-Onset PoAF

Two hundred and thirty-six patients developed new-onset AF after surgery (16.8%). AF occurred at a median of 2 days (1, 4 days) with a median duration of 2 days (1, 4 days). Among the 239 patients with PoAF, 135 (56.48%) patients had immediate new-onset AF postoperatively [86 (35.9%) on day 1, 49 (20.5%) on day 2 postoperatively]. One hundred and sixty patients (66.9%) developed paroxysmal AF, 53 (22.1%) had persistent AF, and 26 (10.8%) had an undocumented type of PoAF.

### 3.3. Early Outcomes

Overall operative mortality was 4.2% (*n* = 59) and was approximately three times significantly higher in patients diagnosed with PoAF (8.9% vs. 3.3%, *p* < 0.001). The stroke rate was significantly higher in the PoAF group (6.9% vs. 1.5%, *p* < 0.001). A higher ratio for necessitating IABP and ECMO support was significantly higher in patients with PoAF (IABP: 9.4% vs. 3.2%, *p* < 0.001; ECMO: 3.4% vs. 1.5%, *p* = 0.040). Rates of postoperative renal failure requiring dialysis were also significantly higher by four times in the PoAF group (13.6% vs. 3.5%, *p* < 0.001) compared to the patients without PoAF. The median ICU stay of PoAF patients was 3 days longer than that of patients without PoAF (*p* < 0.001). The postoperative hospital stay was 5 days longer in PoAF patients (20 days vs. 15 days, *p* < 0.001). Seven patients underwent transcatheter ablation for atrial fibrillation (2.97%) (Table 3).

All patients were discharged on warfarin. Patients with mechanical mitral valves had warfarin for life with a target INR between 2.5 and 3.5. Patients with mitral valve repair or replacement with a bioprosthesis received warfarin for three months. At discharge, 121 PoAF patients received amiodarone (51.5%), 60 (25.5%) received beta-blockers, and 16 (6.8%) received digoxin.

### 3.4. Late Outcomes

**Figure 1:**
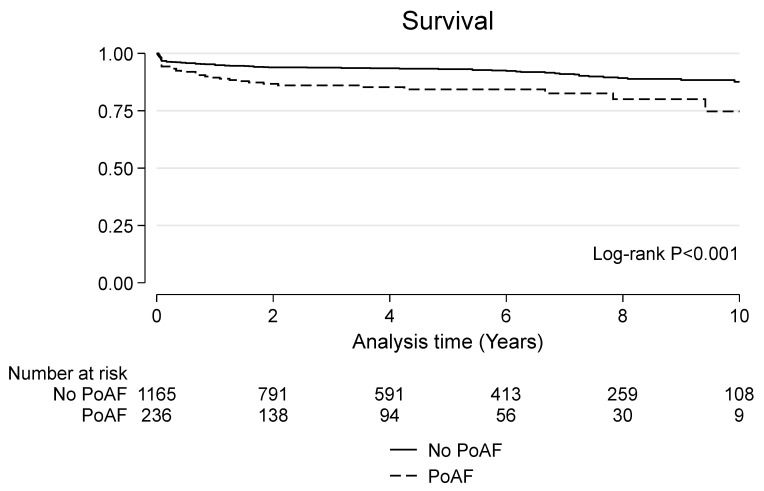
Kaplan-Meier survival curve in patients with and without postoperative atrial fibrillation (PoAF).

**Figure 2:**
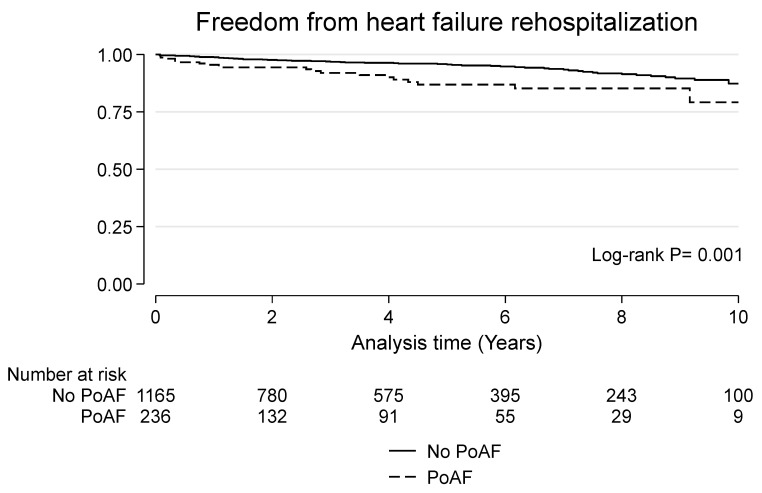
Kaplan–Meier curve for freedom from heart failure rehospitalization in patients with and without postoperative atrial fibrillation (PoAF).

**Figure 3:**
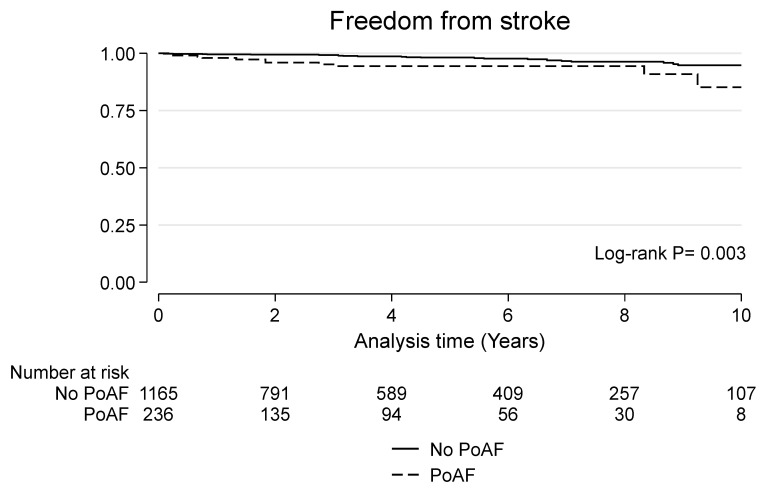
Kaplan–Meier curve for freedom from stroke in patients with and without postoperative atrial fibrillation (PoAF).

**Figure 4:**
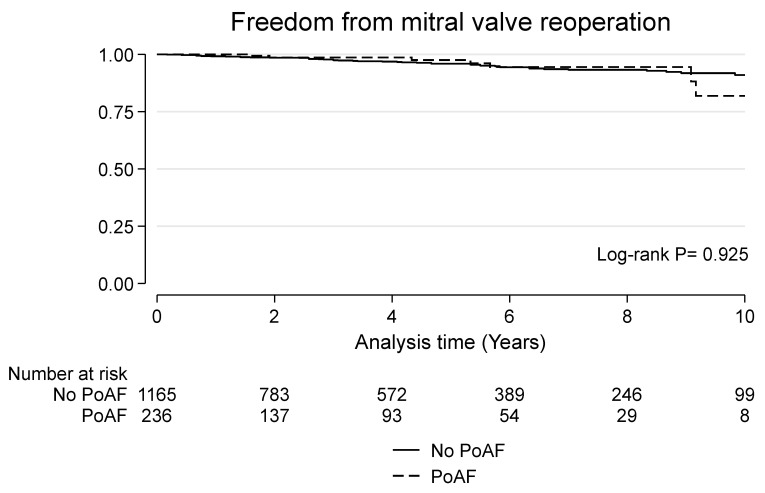
Kaplan–Meier curve for freedom from mitral valve reinterventions in patients with and without postoperative atrial fibrillation.

Follow-up data were available for 1300 patients, and 42 patients were lost to follow-up. The median follow-up duration was 46 (14–87) months. Of the 1342 survivors, 64 patients died at follow-up, with a mean survival time estimate of 138.4 months (95% confidence interval: 135.6–141.3 months). One-, five-, and ten-year freedom from all-cause mortality were 94% ± 0.7, 91.7% ± 0.8, and 85.7% ± 1.6, respectively. Freedom from all-cause mortality was significantly better in patients without PoAF than in those with PoAF (log-rank *p* < 0.001). The one-, five- and ten-year survival rates of patients without PoAF were 94.9% ± 0.7, 93.1% ± 0.8, and 87.6 ± 1.6, respectively, while in PoAF patients, they were 89.5% ± 2.0, 84.3 ± 2.7, and 74.7% ± 6.3, respectively (Figure 1). Eighty patients required rehospitalization for heart failure with one-, five-, and ten-year freedom from rehospitalizations of 98.2% ± 0.4, 94.3% ± 0.8, and 86.2% ± 1.9, respectively. Patients who developed PoAF had significantly higher rehospitalization for heart failure (log-rank *p* = 0.001) (Figure 2). Stroke occurred in 36 patients. Overall, freedom from stroke was 99.2% ± 0.3 at one year, 97.5% ± 0.5 at five years, and 93.6% ± 1.3 at ten years. Freedom from stroke was significantly lower (log-rank *p* = 0.003) in the PoAF group. The freedom from stroke events of patients with and without PoAF was 97.9% ± 1.0 vs. 99.5% ± 0.2 at one year, 94.4% ± 1.9 vs. 98.1% ± 0.5 at five years, and 85.2% ± 6.6 vs. 94.8 ± 1.2 at ten years, respectively (Figure 3). Mitral valve reintervention was required in 54 patients; 2 underwent transcatheter mitral valve-in-valve implantation, while 52 underwent redo mitral valve surgery. Freedom from mitral valve reinterventions at one, five, and ten years was 99.3% ± 0.2, 96.2% ± 0.7, and 90.3% ± 1.6, respectively. Mitral valve reintervention was not significantly different between groups (log-rank *p* = 0.925). Freedom from mitral valve reintervention in patients without and with PoAF was 99.2% ± 0.3, 96.0% ± 0.7, 91.0% ± 1.6 vs. 100%, 97.5% ± 1.5, and 81.9% ± 8.6, respectively (Figure 4).

### 3.5. Multivariable Analysis

PoAF was significantly associated with an increased risk of mortality (hazard ratio (HR): 1.613 (95% CI: 1.048–2.483); *p* = 0.03), heart failure rehospitalization (HR: 2.156 (95% CI: 1.276–3.642); *p* = 0.004), and stroke (HR: 2.722 (95% CI: 1.321–5.607); *p* = 0.007). However, PoAF was not associated with increased mitral valve reinterventions (HR: 0.938 (95% CI: 0.422–2.087); *p* = 0.875) (Table 4).

## 4. Discussion

PoAF remains one of the most common complications after cardiac surgery, with a reported incidence of up to 40% [14,15]. Several studies reported that PoAF negatively affected short-term outcomes after cardiac surgery; however, few studies reported the long-term effects of PoAF, and these studies included mainly CABG or mixed cardiac surgery [16]. The characteristics of patients undergoing mitral valve surgery in our region are unique because of the high prevalence of rheumatic fever [17]. Mitral valve surgery is one of the most common cardiac procedures performed in our institution [10,18,19]. Studies reporting the long-term effects of PoAF after mitral valve surgery are scarce [20]. This cohort study evaluated the incidence of PoAF and its short- and long-term effects in patients undergoing mitral valve surgery. The reported incidence of PoAF was 16.8%. This incidence is lower than that reported in the literature [20], which could be attributed to younger patient age, a smaller proportion of patients with renal impairment, and end-stage renal disease. Furthermore, PoAF was defined as any episode requiring treatment, and transient episodes may not be detected. In this study, the preoperative creatinine clearance was significantly lower in PoAF patients than in non-PoAF patients (98.1 (70.4, 126) vs. 85.8 (52.7, 106.7) mL/min, *p* < 0.001). This creatine clearance was significantly higher than what was reported by Bramer and colleagues (73.1 ± 26 vs. 66.9 ± 27 mL/min, *p* = 0.001) in patients without PoAF vs. with PoAF after mitral valve surgery, respectively [20]. Ahlsson and colleagues reported a 28.9% incidence of postoperative AF after CABG, with no difference in creatinine levels between non-PoAF and PoAF patients (99.4 ± 69.1 vs. 109.8 ± 78.5 µmol/L, *p* = 0.117) [21]. We reported lower creatinine levels in our cohort compared to the Ahlsson et al. study with higher creatinine levels in PoAF patients [77 (62, 93) vs. 81 (67, 112), *p* < 0.001]. Al-Shaar and colleagues reported an incidence of 22.12% of PoAF after CABG [22]. They reported a 3.9% prevalence of preoperative renal failure in the matched cohort, slightly higher than that reported in our series (3.4%). Attaran and associates reported an incidence of PoAF of 28%, and the prevalence of chronic kidney disease in their matched cohort was 6.3% [23].

Another major finding of this study is that PoAF after isolated or concomitant mitral valve surgery was associated with lower long-term survival and a higher probability of heart failure rehospitalization and stroke. These findings support the hypothesis made by Goyal et al. in their retrospective cohort analysis that PoAF is related to incident heart failure hospitalization among patients undergoing cardiac operations who had no prior history of heart failure [24]. These findings support the prognostic significance of PoAF and raise the possibility that it can be used to identify patients with subclinical heart failure and those at high risk of developing the condition [24]. Additionally, our results concur with those of Lin et al., who reported that PoAF was linked to an increased risk of stroke and mortality in their meta-analysis [25]. Wang et al. reported that PoAF after cardiac surgery was associated with an increased risk of short- and long-term stroke [26]. Bramer and colleagues found that PoAF negatively affected long-term survival in patients undergoing mitral valve surgery [20]. Another meta-analysis reported a significant association between PoAF after cardiac surgery and lower survival and stroke (16).

PoAF was linked to greater early mortality rates, ranging from 2.4 to 7.4% after cardiac surgery [25,27]. Our findings revealed that the total operative mortality was 4.2% and that the risk was roughly three times higher in patients with newly diagnosed PoAF. Furthermore, there was an association between PoAF and increased postoperative stroke rate, dialysis, and longer ICU and hospital stay. Therefore, PoAF was associated with poor short- and long-term survival.

This study indicated that patients who developed PoAF after mitral valve surgery should be considered at high risk for long-term complications such as stroke and heart failure rehospitalization. Therefore, close clinical follow-up and monitoring of these patients and probably specific interventions such as medical and transcatheter therapy should be considered in this group of patients.

### 4.1. Study Implications

The study identified that PoAF was associated with lower survival rates and higher rates of heart failure rehospitalization and stroke. These patients may benefit from several measures. Future studies are required to identify high-risk patients who are more prone to developing PoAF. Those patients may benefit from prophylactic antiarrhythmic therapy, either preoperatively or immediately postoperatively [28]. Furthermore, patients who develop PoAF require close follow-up after discharge to monitor and prevent the development of future complications. These interventions require future research to evaluate their effects on the patient’s long-term outcomes [29]. Implementing telemedicine technology could improve patient outcomes, and virtual clinics have shown efficacy in several conditions, such as anticoagulation management [30].

### 4.2. Study Limitations

The study is limited by the retrospective design, and several risk factors could have affected the outcomes and were not included in the study. PoAF was defined as episodes requiring treatment, and several episodes could have passed unnoticed. The temporal relationship between PoAF and other postoperative complications was not assessed, and it is not known whether PoAF increased the complication rates or if the reverse could be true. Furthermore, data about postdischarge management of PoAF were not captured in the study, and the diagnosis of AF was limited to the postoperative hospital stay period. Last, the study is a single-center experience, and the generalization of the results needs further study.

## 5. Conclusions

Atrial fibrillation after mitral valve surgery is a common complication, with an increased risk of operative mortality and hospital complications. PoAF was associated with lower long-term survival, increased heart failure rehospitalization, and stroke. Future studies are needed to evaluate strategies that can be implemented to improve the outcomes of these patients.

## Figures and Tables

**Table 1:** Baseline demographic and clinical characteristics.

Characteristic		ALL Patients (*n* = 1401)	No PoAF (*n* = 1165)	PoAF (*n* = 236)	*p* Value
**Age, y**		56 (44, 64)	55 (43, 63)	62 (49.5, 70)	<0.001
**Weight, kg**		74.4 (64, 82.4)	74 (64, 82.4)	75.1 (65,82)	0.746
**Gender**					0.798
**Male**		773 (55.2)	641 (55)	132 (55.2)	
**BMI (kg/m^2^)**		27.9 (24.6, 31.5)	27.8 (24.5, 31.5)	28.5 (25.2, 32)	0.121
**MI**		268 (19.1)	219 (18.8)	49 (20.8)	0.484
	<24 h	30 (2.3)	22 (2)	8 (3.7)	
	Within 30 days	77 (5.9)	66 (6)	11 (5)	
	1–3 months	29 (2.2)	23 (2.1)	6 (2.7)	
	>3 months	47 (3.6)	40 (3.6)	7 (3.2)	
**Dialysis**		48 (3.4)	33 (2.8)	15 (6.4)	0.005
**Extracardiac arteriopathy**		28 (2)	21 (1.8)	7 (3)	0.239
**Poor mobility**		43 (3.1)	31 (2.7)	12 (5.1)	0.049
**Previous cardiac surgery**		167 (12.1)	133 (11.6)	34 (14.6)	0.194
**Smoking**		107 (7.6)	96 (8.2)	11 (4.7)	0.059
**History of congestive heart failure**		563 (40.19%)	459 (39.4%)	104 (44.07%)	0.182
**Stroke**		57 (4.1)	44 (3.8)	13 (5.5)	0.219
**Hypertension**		671 (47.9)	552 (47.4)	119 (50.4)	0.394
**COPD**		45 (3.2)	35 (3)	10 (4.3)	0.320
**Active endocarditis**		47 (3.4)	37 (3.2)	10 (4.3)	0.401
**Critical preoperative state**		40 (2.9)	32 (2.8)	8 (3.4)	0.580
**DM**		606 (43.3)	505 (43.3)	101 (42.8)	0.876
**Creatinine μmol/L**		77 (63, 95)	77 (62, 93)	81 (67, 112)	<0.001
**Creatinine Clearance (mL/min)**		95.9 (67.1, 124.1)	98.1 (70.4, 126)	85.8 (52.7, 106.7)	<0.001
**NYHA**					0.906
	III	858 (61.8)	716 (62.2)	142 (60.2)	
	IV	143 (10.3)	116 (10.1)	27 (11.4)	
**EuroSCORE II**		2.52 (1.36, 5.43)	2.4 (1.3, 5.2)	3.4 (1.4, 7.5)	<0.001
**IABP**		21 (1.5)	10 (0.9)	11 (4.7)	<0.001
**Inotropes**		207 (14.8)	151 (13)	56 (23.8)	<0.001
**Ventilation**		176 (12.6)	132 (11.3)	44 (18.7)	0.002

For continuous variables, mean ± SD or median (25th, 75th percentile); for categorical variables, *n* (%). PoAF: postoperative atrial fibrillation; BMI, body mass index; MI, myocardial infarction; COPD, chronic obstructive pulmonary disease; DM, diabetes mellitus; NYHA; New York Heart Association; IABP, an intra-aortic balloon pump.

**Table 2:** Intraoperative data of mitral valve surgery by postoperative atrial fibrillation.

Characteristic		All Patients (*n* = 1401)	No PoAF (*n* = 1165)	PoAF (*n* = 236)	*p* Value
**Status**					0.026
	Elective	1345 (96.1)	1124 (96.6)	221 (93.6)	
	Urgent	15 (1.1)	13 (1.1)	2 (0.8)	
	Emergency	40 (2.9)	27 (2.3)	13 (5.5)	
**Concurrent CABG**		573 (40.9)	473 (40.6)	100 (42.4)	0.614
**Type of MV surgery**					0.029
	RepRepair	738 (52.7)	629 (54)	109 (46.2)	
	Replacement	663 (47.8)	536 (46.01)	127 (53.8)	
**Concurrent TV surgery**					0.155
	Repair	519 (37)	429 (36.8)	90 (38.1)	
	Replacement	40 (2.9)	29 (2.5)	11 (4.8)	
**CPB (min)**		132 (101, 164)	130 (98, 163.5)	139 (108,165)	0.039
**Cross clamp (min)**		101 (75, 127)	100 (75, 126)	109 (78, 133)	0.025

For continuous variables, mean ± SD or median (25th, 75th percentile); for categorical variables, *n* (%). PoAF: postoperative atrial fibrillation; CABG, coronary artery bypass graft; MV, mitral valve; TV, tricuspid valve; CPB, cardiopulmonary bypass.

**Table 3:** Postoperative data after mitral valve surgery by postoperative atrial fibrillation.

Characteristic				
	All Patients (*n* = 1401)	No PoAF (*n* = 1165)	PoAF (*n* = 236)	*p* Value
**Early IABP**	59 (4.2)	37 (3.2)	22 (9.4)	<0.001
**Early ECMO**	25 (1.8)	17 (1.5)	8 (3.4)	0.040
**Early CRRT/Dialysis**	73 (5.3)	41 (3.5)	32 (13.6)	<0.001
**Early stroke**	33 (2.4)	17 (1.5)	16 (6.9)	<0.001
**ICU stay, d**	3 (1, 5)	2 (1, 4)	5 (2, 11)	<0.001
**Hospital stay, d**	16 (11, 28)	15 (11, 25)	20 (14, 36)	<0.001
**Hospital mortality**	59 (4.2)	38 (3.3)	21 (8.9)	<0.001

For continuous variables, mean ± SD or median (25th, 75th percentile); for categorical variables, *n* (%). PoAF: postoperative atrial fibrillation; IABP, intra-aortic balloon pump; ECMO, extracorporeal membrane oxygenation; CRRT, Continuous renal replacement therapies.

**Table 4:** Multivariable Cox regression for factors affecting survival, stroke, heart failure rehospitalization, and mitral valve reoperation.

Survival	HR (95% CI)	*p* Value
EuroSCORE II	1.034 (1.019–1.048)	<0.001
Age	1.030 (1.012–1.048)	0.001
Dialysis	2.852 (1.602–5.075)	<0.001
Concomitant tricuspid valve surgery	2.070 (1.512–2.834)	<0.001
Preoperative ventilation	2.307 (1.371–3.880)	0.002
Diabetes mellitus	2.076 (1.336–3.225)	0.001
Preoperative intra-aortic balloon pump	2.697 (1.249–5.820)	0.012
Previous myocardial infarction	1.607 (1.066–2.424)	0.024
Postoperative atrial fibrillation	1.613 (1.048–2.483)	0.030
**Stroke**		
Smoking	4.626 (1.984–10.785)	<0.001
Postoperative atrial fibrillation	2.722 (1.321–5.607)	0.007
Diabetes mellitus	2.609 (1.291–5.271)	0.008
Previous stroke	3.241 (1.104–9.515)	0.032
**Heart failure rehospitalization**		
Combined CABG	1.901 (1.109–3.261)	0.020
Preoperative intra-aortic balloon pump	3.791 (1.325–10.847)	0.013
Hypertension	2.325 (1.310–4.128)	0.004
Postoperative atrial fibrillation	2.156 (1.276–3.642)	0.004
EuroSCORE II	1.025 (1.004–1.047)	0.021
Diabetes mellitus	1.732 (1.016–2.952)	0.044
**Mitral valve reintervention**		
Postoperative atrial fibrillation	0.938 (0.422–2.087)	0.875
Female	1.901 (1.049–3.445)	0.034
Concomitant CABG	0.414 (0.201–0.853)	0.017
Dialysis	5.587 (1.676–18.627)	0.005

CABG: coronary artery bypass grafting; CI: confidence interval; HR: hazard ratio.

## Data Availability

Due to the nature of the research, and due to the hospital protocol, supporting data is not available.
